# Self-esteem in the deaf who have become cochlear implant users as adults

**DOI:** 10.1371/journal.pone.0203680

**Published:** 2018-09-11

**Authors:** Joanna Kobosko, W. Wiktor Jedrzejczak, Elżbieta Gos, Anna Geremek-Samsonowicz, Maciej Ludwikowski, Henryk Skarzynski

**Affiliations:** 1 Institute of Physiology and Pathology of Hearing, Warsaw, Poland; 2 World Hearing Center, Kajetany Nadarzyn, Poland; University of Texas at Dallas, UNITED STATES

## Abstract

**Objective:**

Self-esteem is a good predictor of mental health and is crucial for well-being and psychological functioning. It is especially important in situations where there are potential mental health problems, such as in people suffering from hearing loss or total deafness. This study aims to gauge the level of self-esteem in adults with hearing problems, in particular those who, in adulthood, had received a cochlear implant (CI). The subjects had different onset (pre-lingual/post-lingual) and amount (deafness/partial deafness) of hearing loss, and their current level of self-esteem was compared to that of the general population. The association of self-esteem with other deafness-related variables (e.g. satisfaction with their CI or whether they also used a hearing aid) and sociodemographic factors was also investigated.

**Methods:**

Data were obtained from questionnaires mailed to patients who, when adult, had received a CI. The subjects were divided into four subgroups: subjects with pre-lingual deafness, post-lingual deafness, pre-lingual partial deafness, and post-lingual partial deafness. To evaluate their self-esteem, the Rosenberg Self-Esteem Scale (RSES) was used. For data on sociodemographic status and information related to deafness and CI, we used our own Information Inquiry form. For statistical analysis of the results, we compared means (*t*-test, ANOVA), investigated correlations, and applied linear regression.

**Results:**

The self-esteem of deaf and partially deaf CI users was significantly lower than in the general population, especially for post-lingually deafened subjects. The only factor related to deafness and CIs that explained self-esteem was self-rated satisfaction with the CI–meaning that higher satisfaction was associated with higher self-esteem. The major sociodemographic factor that explained self-esteem was marital/partnership status (being in a relationship was helpful). Also men had higher self-esteem than women. Those with higher levels of education, and those working or studying, had higher self-esteem than those who did not. RSES was found to have a single-factor structure.

**Conclusion:**

Deafness and partial deafness appear to be risk factors for lower self-esteem, a finding that rehabilitation, medical, educational, and employment communities should be made aware of. Medical intervention in the form of a CI supplies the person with improved hearing, but it is not a panacea: their self-esteem is still vulnerable, and reinforcement of self-esteem is an aspect that professionals should focus on. Psychological, psycho-educational, and psychotherapeutic interventions have important roles to play for CI recipients.

## Introduction

### Self-esteem

Self-esteem, according to Rosenberg [1,2] is an emotional attitude toward oneself and one’s own competencies. It is a kind of global self-assessment. Rosenberg says that high self-esteem means that one considers oneself as "good enough", a valuable person, whereas low self-esteem means that one is dissatisfied with oneself, an object of rejection. In order to evaluate global self-esteem, the Rosenberg Self-Esteem Scale (RSES) was created, a scale that has become highly popular [1–3].

RSES has commonly been used to measure global self-esteem in the deaf and hard of hearing (DHH) population [4–9]. It has also been adapted to sign language, e.g. in the United States [4] and Israel [7]. There are many variables that affect self-esteem. It increases with age up to 50–60 years and then decreases (for a review see [10]); is higher in people with better education [11], higher with better quality partner/marital relationships [12], and lower in the unemployed [13]. On the other hand, gender seems to have no effect on self-esteem [10,13], although there are studies which show a slightly higher self-esteem in men [10,14].

### Outline of the current study

The present study on global self-esteem concerns adults with deafness or partial deafness. For our purposes, deaf adults are considered here to be those whose hearing loss meets the criteria of severe or profound hearing loss [15]. Adults with partial deafness are considered to be those who have normal hearing (or at least only moderate hearing loss) in the low frequency range but severe or profound hearing loss at high frequencies [16]. Usually, the hearing of both of these groups of individuals is so degraded that hearing aids (HAs) may not provide appropriate and adequate auditory benefit. A cochlear implant (CI) then becomes a viable option to overcome the problem [17]. A CI is an electronic hearing prosthesis whose main part is surgically implanted into the cochlea and bypasses nonfunctional parts of the inner ear. It transforms sound into electric signals that in turn stimulate the auditory nerve. As a result, a person with a CI is able to hear sounds and most subjects can understand speech, although comprehension via a CI is still far less than with natural hearing. Nevertheless, despite its limitations, it has been well documented that CIs improve hearing and quality of life [18–21]. In Poland, the first CIs were obtained by adults with post-lingual deafness in 1992 [22]; one year later they were given to adults with pre-lingual deafness [23]; and since 2002, adults with partial deafness have been implanted [24].

In many studies of global self-esteem, the concept has been associated with how well people function in the psychosocial sphere [10, 25–26]. It has been empirically confirmed that, at least in the general population, self-esteem is a good mental health predictor [26]. Likewise, in a heterogeneous population of deaf or hard of hearing (DHH) individuals, it has also been shown to be an important indicator of mental health and general quality of life [5, 27–28].

### Pre-lingual and post-lingual hearing loss

Pre-lingual deafness is usually associated with childhood difficulties in developing speech and language [29–34]. Surprisingly, however, these same difficulties can also often become apparent with sign language skills, especially when deaf children live in the world of hearing families [35–36]. It seems that, for whatever reason, the majority of deaf people are susceptible to a problem in communicating effectively in a social (hearing) environment, so that impaired psychosocial functioning can in turn lead to social rejection, stigmatization and labeling, low-status jobs, and low economic status–all of which can adversely affect their self-esteem [9, 37–39]. For a deaf child to develop psychologically and thrive socially, including in terms of their self-esteem, the family context is important [40–41]. As expected, their day-to-day social milieu plays a significant role, and development depends on whether they are mostly immersed in a hearing environment or a Deaf environment, noting that the Deaf culture and its sign language constitutes a linguistic minority [4, 35,37,39,42–43].

In the case of people with acquired deafness, i.e. those that are post-lingually deaf, hearing can be lost suddenly or gradually, but in either case the loss is often traumatic [8, 44–45]. Deafness is therefore looked upon as a severe handicap and loss [27], and it becomes a stressful life experience that can trigger negative changes in self-esteem [46]. Those who were not born DHH face different problems: they have no choice but to adapt to the disability, knowing for certain that their hearing has been irrevocably lost and that they must learn to live as a deaf person and find effective strategies to communicate in social interactions, either professionally or in family roles. They know no other circumstance and are forced to develop a new personal identity: including deafness into it as an optimal solution. It appears that the trauma undergone by people with post-lingual deafness is more conducive to lowered self-esteem [47–48].

### Self-esteem and hearing loss

A meta-analysis of self-esteem in the DHH population in the 1990s concluded that self-esteem was lower than, or perhaps similar to, those with normal hearing [42]. However, a number of methodological deficiencies make it difficult to compare the results of such self-esteem studies (e.g., the use of self-esteem tools that do not take account of the specific language and communication modes of the DHH, and the large heterogeneity of the subjects–not only in the degree and etiology of deafness but also in the different family, educational, social, and socioeconomic status). The results of subsequent studies in adults with pre-lingual deafness also appear inconsistent in terms of self-esteem ratings in specific domains [49–50] or assessed globally [7,9,51–53]. However, the general finding is that people with post-lingual deafness have lower global self-esteem compared to normally hearing people [8,27], although there are still only a limited number of studies on pre-lingually deaf adults [54]. For DHH CI users, the studies are even fewer (e.g. [55]).

Of particular interest, previous studies found that deaf adults whose parents used to communicate with them using sign language had generally higher self-esteem than those who used oral communication [5]. The latter, typically forced to use lip reading, experienced a sense of isolation and exclusion from family conversations, which often had negative effects on their self-esteem [38]. Notwithstanding, there are also some other findings on deaf adolescents and young adults which showed no effect from how the family communicated [56]. In terms of the mental health of deaf adolescents, some authors point to the important role of the (hearing) mother–(deaf) child interaction [57]. Taken together, satisfactory communication at home can be seen as a good predictor of self-esteem [58].

In initial studies on DHH, it was argued that social benefits, including better self-esteem, were provided by education in schools for the deaf, even though integrated schools provide academic advantages [59]. Later studies indicate that higher levels of global and domain-specific self-image emerge from mainstreamed DHH adolescents [58,60]. Some others conclude that the self-esteem of deaf people bears no relationship to the type of education [38,42].

When considering the relationship between self-esteem and various deafness-related factors, the role of Deaf identity or Deaf acculturation also needs to be taken into account. Research shows that deaf people who are culturally Deaf but who also have bicultural identities appear to show higher self-esteem [38,61–62], while those with marginal identity score at lower levels [62]. Lower self-esteem also seems to be associated with marginal acculturation [5,63], while a study by Hintermair [5] found that hearing acculturation in DHH people was positively correlated with self-esteem.

On the other hand, many factors *do not* play a role in deaf people’s self-esteem: whether the person communicates in sign language or speech (or bilingually–speech and sign language) does not appear to matter; similarly, neither does age or sex [4–5,9,38,56]. However, education has been shown to be a good predictor of self-esteem in adults with varying degrees of hearing loss, even when they used a variety of communication modalities (25% of those interviewed used only oral communication) [9].

One would think that the degree of hearing loss would be a major factor in self-esteem, but findings indicate that the degree of hearing loss is unrelated to psychological distress (taking note that distress is itself negatively correlated with self-esteem of DHH subjects) [27,64]. In just a few studies, the level of mental distress in DHH patients was compared between pre- and post-lingual groups: the result was that the post-lingual group had higher distress compared to pre-lingual patients [27], suggesting that a similar relation might hold in terms of global self-esteem. There are also results indicating that people with only mild hearing loss have worse psychosocial functioning than the profoundly deaf [65–66] or those with normal hearing [67], suggesting they have lower self-esteem compared to these groups.

### Rationale for the present study

In view of the previous findings mentioned above, there is much about self-esteem that still needs to be clarified, particularly when the CI factor is added. Few studies focus on the global self-esteem of DHH adults in general, and research on the self-esteem of DHH individuals who have opted for a CI is scant e.g. [8,54]. Indeed, it appears rather strange that in a time when CIs are now common and there has been much research aimed at improving the quality of life of this group of deaf adults [18–21, 68–69], studies have not directly focused on global self-esteem. Indirectly, of course, they suggest that a CI enhances global self-esteem, but hard evidence is lacking. Empirical research on the global self-esteem of DHH people will give us the data needed to frame future research and provide the best possible interventions. We should always keep in mind that despite the technological advances in medicine, the different types of hearing implants, and the diverse patient groups now eligible for a CI, deafness and hearing loss remain risk factors to mental health.

### Aim of the study

The purpose of the study was to assess the global self-esteem of deaf and partially deaf subjects who have used one CI from adulthood. To achieve this, the RSES was used in its Polish adaptation [1–3]. In particular, we wanted to explore how self-esteem connects with certain variables related to deafness and CI: hearing loss (deafness or partial deafness); pre- or post-lingual hearing impairment; duration of CI use; configuration of hearing prosthesis (CI or CI + HA); satisfaction with CI; and sociodemographic variables such as gender, age, education, partnership or marital status, and employment or learning status. Finally, we wanted to investigate the methodological question of whether the RSES structure is unidimensional or two-dimensional (with positive and negative items), as there are some different views on this matter (e.g. [2–3]).

## Material and method

### Participants

The study group consisted of 120 CI users aged 22 to 60 years old. Sociodemographic information on them is presented in Table 1. All participants had been CI users since adulthood, i.e. after the age of 18, and they had only a single CI. Some of them used a HA in the non-implanted ear. Half the subjects had pre-lingual hearing loss and the other half had post-lingual hearing loss. Those with pre-lingual deafness were either born deaf (congenital hearing loss) or became deaf prior to speech and language acquisition. The subjects with post-lingual deafness lost their hearing after they had developed speech (over 3 years of age). Within each subgroup, 30 were deaf and 30 had partial deafness. Deafness is defined here as severe or profound hearing loss at all pure tone audiometric frequencies (>70 dB HL at frequencies 0.25–8 kHz); partial deafness is defined as severe or profound hearing loss at audiometric frequencies above 1 kHz, with at least 70 dB HL threshold from 0.25 to 1 kHz. Subjects with partial deafness experience significant difficulties in comprehending speech, even though they hear the sound of it [68, 70–71]. The result is that they often encounter barriers in communicating with people, and fail to respond in a social situation. They often have a sense of life "on the border" of two worlds: deaf and hearing.

**Table 1:** Sociodemographic data, data related to deafness and CI use, and Cronbach’s α for the Rosenberg Self-Esteem Scale of study participants.

		Deafness			Partial deafness			All participants
		Pre	Post	Total	Pre	Post	Total	
*N*		30	30	60	30	30	60	120
Age (yr)	Mean (SD)	35.73 (9.93)	42.43 (11.19)	39.01 (11.02)	37.73 (12.78)	44.40 (10.24)	41.07 (11.97)	40.08 (11.50)
	Range	23–59	23–60	23–60	22–60	23–59	22–60	22–60
Gender	Male (%)	50.0	50.0	50.0	50.0	50.0	50.0	50.0
	Female (%)	50.0	50.0	50.0	50.0	50.0	50.0	50.0
Educational status	Primary or secondary (%)	66.7	83.3	75.0	73.3	63.3	68.3	71.7
	Diploma or university (%)	33.3	16.7	25.0	23.3	26.7	25.0	25.0
	No response (%)	-	-	-	3.3	10.0	6.7	3.3
Marital (partnership) status	In relationship (%)	43.3	43.3	43.3	46.7	56.7	51.7	47.5
	Not in relationship (%)	56.7	56.7	56.7	53.3	40.0	46.7	51.7
	No response (%)	-	-	-	-	3.3	1.7	0.8
Employment (or study) status	Employed (%)	63.3	63.3	63.3	53.3	56.7	55.0	59.2
	Unemployed (%)	36.7	36.7	36.7	30.0	33.3	31.7	34.1
	No response (%)	-	-	-	16.7	10.0	13.3	6.7
CI experience (years)	Mean (SD)	4.23 (1.99)	3.60 (1.99)	3.92 (2.00)	4.07 (2.26)	5.11 (2.78)	4.59 (2.56)	4.24 (2.31)
	Range	2–6	2–6	2–6	1–10	1–9	1–10	1–10
Configuration of hearing prosthesis	CI (%)	26.7	50.0	38.3	30.0	66.7	48.3	43.3
	CI+HA (%)	73.3	50.0	61.7	66.7	33.3	50.0	55.9
	No response (%)	-	-	-	3.3	-	1.7	0.8
CI satisfaction (%)	Mean (SD)	76.54 (20.83)	77.14 (24.21)	76.84 (22.40)	77.40 (17.27)	83.05 (16.84)	80.12 (17.15)	78.43 (20.02)
	Range	23.60–100.0	15.53–100.0	15.53–100.0	33.54–100.0	45.34–100.0	33.54–100.0	15.53–100.0
Cronbach’s α		0.864	0.906	0.872	0.879	0.875	0.886	0.879

Data related to deafness and CI use was also recorded: time of onset of hearing impairment (pre-lingual/post-lingual); amount of hearing loss (deafness/partial deafness); years of CI experience; configuration of hearing prosthesis (CI or CI+HA); and satisfaction with their CI (Table 1). Satisfaction with their CI is defined as the subjective state of the CI user which reflects the overall feeling of benefit (audiological and nonaudiological) attributable to their CI, including quality of life and general psychological well-being [8].

As a reference, a group with normal hearing (1121 persons aged 15 to 55 years) and tested with the Polish adaptation of RSES was used [2].

Data on the duration of hearing loss was also collected for post-lingual subjects (*M* = 29.1 years, *SD* = 13.8, range = 1–56) and age at the time of implantation (*M* = 35.9 years, *SD* = 11.8, range = 18–58). The duration of hearing loss in pre-lingual subjects was identical to age, and age at the time of implantation was very strongly correlated with age (*r* = 0.97, *p* <0.001).

It should also be emphasized that the study group did not include pre-lingually deaf people who were raised primarily with sign language in a Deaf environment; neither did it include people with a bilingual education (where the deaf child simultaneously acquires both spoken language and sign language) who are still recognized in Poland as members of the deaf population, even if they have a CI. The subjects here did not know sign language (there was a specific question on this in the survey form), they had completed mainstream or integrated education with hearing persons at various levels, and, as can be inferred indirectly, they had hearing acculturation. In terms of language and communication, they used only spoken Polish. They were therefore a homogeneous group which can be considered audiologically deaf (but not culturally Deaf or bicultural). Subjects who acquired a CI during childhood or adolescence were also not included, because such people are young, and the present study wanted to focus on subjects in early to middle adulthood (18 to 60 years).

### Study procedure

All procedures were approved by the ethics committee of the Institute of Physiology and Pathology of Hearing, Warsaw (approval number: IFPS:/KB/02/2014). No written consent was provided, since the study was based on anonymous questionnaires.

A survey form was used to collect information on sociodemographic variables (gender, age, education, employment or study status, partner or marital status), and variables related to deafness and CI, such as when hearing loss occurred and how long a CI had been used. One question asked about satisfaction with the CI, and the respondents marked the extent to which they were satisfied with their CI on a visual analog scale (a line with no markings except at the ends: 0–dissatisfied; 10–very satisfied). The line, 161-mm long, fitted across the page of the form. The respondent’s response was measured in millimeters and converted to a percentage. This variable did not meet the assumptions of a normal distribution and was converted to a logarithm. More about the method of measuring satisfaction with a CI can be found elsewhere [8]. Finally, subjects also received a RSES form [1–2], described below.

The research was conducted by mail. Respondents received a letter requesting anonymous and voluntary participation, questionnaires, and a survey form. The study was conducted in two stages: the first reached out to people with pre- or post-lingual deafness, for which the response rate was 64.0%. The second stage was addressed to subjects with partial deafness, for which there was a very similar response rate (63.5%).

### Rosenberg’s Self-Esteem Scale (RSES)

The Rosenberg Self-Esteem Scale (RSES) [1]; in Polish adaptation [2], measures global self-esteem, which is considered a relatively enduring feature. It consists of 10 items, 5 formulated positively (#1, 3, 4, 7, 10), e.g. “I take a positive attitude toward myself”; and 5 framed negatively (#2, 5, 6, 8, 9), e.g. “All in all, I am inclined to feel that I am a failure”. The scale is assumed to be one-dimensional, as was the case in its Polish adaptation [2]. However, some researchers, as a result of their analyses, believe that RSES has a bi-factor solution, where one factor is positive and the other negative [3,72]. Confirmatory factor analysis (CFA) was applied to our dataset to investigate the goodness-of-fit of a bi-factor model in which two factors grouping positive and negative items were assumed. The bi-factor model did not show a good fit to the data: *χ*^2^(34) = 87.42; *p*<0.001; CFI = 0.89; NFI = 0.84; RMSEA = 0.12. According to Hu and Bentler [73] a good model fit should provide insignificant *χ*^2^-values. For CFI, the cut-off value should be 0.90 or greater; for NFI a value above 0.90 is considered acceptable. Good model fit is indicated by an RMSEA value of 0.06 or less. According to these criteria, a bi-factor model for RSES verified in people with hearing loss must be considered inadequate.

In the RSES, respondents consider each statement and indicate to what extent it applies to themselves. It uses a 4-point Likert scale, ranging from strongly agree (4) to strongly disagree (1). RSES scores are therefore in the range of 10 to 40 points, and the higher the score, the higher the self-esteem. Rosenberg considers that high self-esteem means that the subject is convinced that he is "good enough", a valued person.

In the present study, Cronbach alpha for all subjects was 0.88 and in the subgroups with different hearing loss and with different onset (pre- and post-lingual) ranged from 0.86 to 0.91 (Table 1).

## Results

Global self-esteem in the study group ranged from 17 to 40 points (the higher the value, the more positive self-esteem). The mean self-esteem level was *M* = 28.20, standard deviation *SD* = 4.87. The distribution of the variable was normal (*Z* = 0.07, *p* > 0.05).

### Self-esteem in relation to sociodemographic variables

Global self-esteem for subgroups with different sociodemographic characteristics is presented in Table 2. The self-esteem of deaf people with a CI was related to gender, with men having higher self-esteem (*M* = 29.17, *SD* = 4.89) than women (*M* = 27.23, *SD* = 4.69). Self-esteem was also related to education: deaf people with higher education had higher self-esteem (*M* = 30.37, *SD* = 4.46) than people with basic or secondary education (*M* = 27.37, *SD* = 4.74). Marital (partnership) status was another important factor for high self-esteem: deaf people in a relationship had higher self-esteem (*M* = 29.42, *SD* = 4.78) than those who were not (*M* = 27.10, *SD* = 4.75). Self-esteem was also related to occupation: deaf workers (and students) had higher self-esteem (*M* = 29.07, *SD* = 4.96) than non-working (or non-studying) people (*M* = 26.56, *SD* = 4.53). All these differences were significant (*p* <0.05).

**Table 2:** Average results of RSES in relation to sociodemographic data (standard deviations shown in brackets). Data for normally hearing subjects were taken from Dzwonkowska et al. [2].

		Study group	*p*	Normally hearing	*p* (norm vs hearing impaired)
All		28.20 (4.87)			
Gender	Male	29.17 (4.89)	**<0.05**	29.94 (4.26)	NS
	Female	27.23 (4.69)		29.19 (4.28)	**<0.01**
Educational status	Primary or secondary	27.37 (4.74)	**<0.01**		
	Diploma or university	30.37 (4.46)			
Marital (partnership) status	In relationship	29.42 (4.78)	**<0.01**		
	Not in relationship	27.10 (4.75)			
Employment (or study) status	Employed (%)	29.07 (4.96)	**<0.01**		
	Unemployed (%)	26.56 (4.53)			

NS, not significant

As the unemployed were significantly older than the employed, an analysis of covariance taking age into account was also performed, which confirmed that self-esteem was related to employment (or study): *F*(1,109) = 5.76; *p* = 0.018; *e*^2^ = 0.05.

It was also found that self-esteem tended to decrease with age, but the correlation was not significant.

### Self-esteem in relation to hearing status

**Fig 1:**
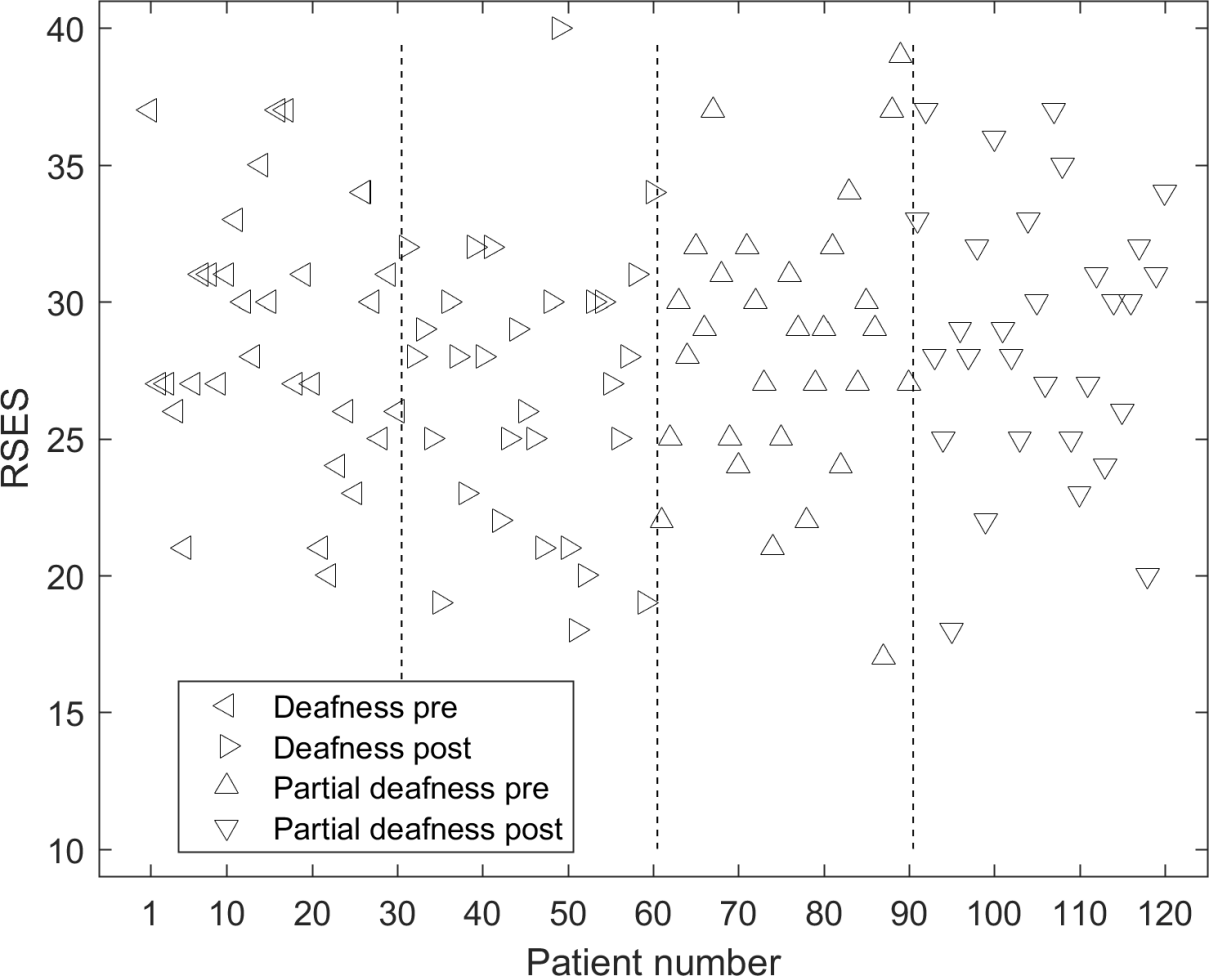
Individual results of RSES for all subjects. The dashed lines separate subgroups with different time of onset of hearing impairment and amount of hearing loss.

Individual results of RSES for all subjects are shown in Fig 1. Table 3 presents descriptive statistics for self-esteem in groups of persons differentiated by time of onset of hearing loss (pre-lingual/post-lingual) and degree of hearing loss (deafness/partial deafness). There were no statistically significant differences between the subgroups (deafness vs partial deafness).

**Table 3:** Average results of RSES in relation to onset and degree of hearing loss (standard deviations shown in brackets). Data for normally hearing subjects were taken from Dzwonkowska et al. [2].

		*N*	Study group	*p*	Normally hearing	*p* (study group vs norm)
Pre-lingual	Deafness	30	28.67 (4.65)	NS	29.49 (4.28)	NS
	Partial deafness	30	28.40 (4.92)		29.49 (4.28)	NS
	Total	60	28.53 (4.75)		29.49 (4.28)	NS
Post-lingual	Deafness	30	26.90 (5.07)	NS	29.49 (4.28)	**<0.01**
	Partial deafness	30	28.83 (4.84)		29.49 (4.28)	NS
	Total	60	27.87 (5.01)		29.49 (4.28)	**<0.01**
Total	Deafness	60	27.78 (4.90)	NS	29.49 (4.28)	**<0.01**
	Partial deafness	60	28.62 (4.84)		29.49 (4.28)	NS
All		120	28.20 (4.87)		29.49 (4.28)	**<0.01**

NS, not significant.

A two-way analysis of variance was performed to test whether self-esteem is related to time of onset and degree of deafness. Time of onset of deafness, the amount of hearing loss, and their interaction, were statistically insignificant.

Self-esteem was positively correlated with satisfaction with the CI: *r* = 0.28; *p* = 0.002. The configuration of the hearing prosthesis (CI vs CI+HA) was not significant for self-esteem. There was also no significant relationship between self-esteem and duration of CI experience.

### Self-esteem in CI users in comparison to those with normal hearing

Dzwonkowska et al. [2] reported data on global self-esteem level in the general Polish population, which makes it possible to compare the self-esteem of people with hearing loss and a CI with those with normal hearing. The results of the comparison are shown in Tables 2 and 3.

In dealing with the sociodemographic data, it was only possible to make comparisons for different genders. Female CI users had lower global self-esteem (*M* = 27.23, *SD* = 4.69) than normally hearing females (*M* = 29.19, *SD* = 4.28), whereas male CI users did not differ in self-esteem from normally hearing males (Table 2).

CI users had significantly lower self-esteem (*M* = 28.20, *SD* = 4.87) than those with normal hearing (*M* = 29.49, *SD* = 4.28), and this difference was primarily due to lower self-esteem in those with profound hearing loss and post-lingual onset (Table 3). Together, these two factors–profound hearing loss and hearing loss obtained post-lingually–are particularly strong in lowering self-esteem.

### The combined effect on self-esteem of sociodemographic factors and variables associated with hearing loss

To summarize the combined effect on self-esteem of sociodemographic factors and variables associated with hearing loss, multiple hierarchical regression was performed. All variables were entered into the model in two blocks: the first was the set of sociodemographic factors, the second was the block containing the variables associated with hearing loss and CI use.

The regression model was statistically significant: *F*(11,87) = 4.15; *p* <0.001. The sociodemographic factors explained 27.9% of self-esteem variance (*R*^2^ adjusted = 24.0%), and after entering the variables associated with hearing loss, the explained self-esteem variance increased to 34.4% (*R*^2^ adjusted = 26.1%). The change in *R*^2^ was not statistically significant: *F*(6,87) = 1.43; *p* = 0.211, so the contribution of predictors associated with hearing loss and CI use to explain variance of self-esteem was rather small. Table 4 shows the outcomes of the regression model.

**Table 4:** Multiple linear regression analysis for RSES of study group.

	*b*	95% confidence interval	SE	β	*T*	*p*
Constant	23.64	17.78; 29.49	2.95		8.02	**<0.001**
Gender	1.55	-0.24; 3.34	0.90	0.16	1.72	0.089
Age	-0.06	-0.17; 0.04	0.05	-0.14	-1.20	NS
Educational status	2.04	-0.05; 4.12	1.05	0.18	1.94	0.055
Marital (partnership) status	3.22	1.37; 5.07	0.93	0.33	3.46	**<0.01**
Employment (or study) status	1.93	0.00–3.87	0.97	0.19	1.99	0.050
Onset of hearing loss	-0.57	-2.63; 1.49	1.04	-0.06	-0.55	NS
Degree of hearing loss	0.05	-1.80; 1.89	0.93	0.01	0.05	NS
CI satisfaction	0.05	0.00; 0.09	0.02	0.19	2.01	**<0.05**
CI experience (years)	0.16	-0.23; 0.55	0.20	0.07	0.81	NS
Configuration of hearing prosthesis	-1.13	-3.02; 0.75	0.95	-0.11	-1.19	NS
Duration of deafness (years)	-0.02	-0.10; 0.07	0.04	-0.04	-0.35	NS

NS, not significant.

In the case of gender, the reference group was women; for education it was primary and secondary; for marital (partnership) status it was not in a relationship; for employment (or study) status it was unemployed. Regression results indicate that marital (partnership) status (presently in a relationship), and satisfaction with CI are significant predictors of self-esteem. They are associated with higher self-esteem, with the greatest impact on self-esteem (highest beta) among CI users being marital (partnership) status. Gender, employment (or study) status, and educational status tended to be significant predictors of self-esteem (for gender *p* = 0.089, for employment status *p* = 0.050, for educational status *p* = 0.055); so being male, being employed, and having better education are associated with higher self-esteem.

## Discussion

The present study has investigated the important psychological underpinnings of global self-esteem in a special population–DHH adults whose hearing loss had different onsets (pre- or post-lingual) and levels (profoundly deaf or partially deaf). All these participants received their CI only after they had become adult.

### Self-esteem and variables associated with deafness and CI use

In overview, the subjects who had been using a CI since adulthood had lower self-esteem compared to the general normally hearing population. This finding corresponds with the existing literature on deafness [7–9, 42, 74–75]. Similar findings have also been found with adolescents [51, 76] and children [77]. In some adult studies, there have been some CI participants, but they were usually dispersed throughout the subject group, not specifically singled out for study [7, 9]. A clear outcome of our study is that the self-esteem of those who lose their hearing post-lingually, and to a profound level, is particularly vulnerable. These people had significantly lower RSES scores (i.e. less positive emotional appreciation of themselves and their competence) than the general population, a finding supported by related research [8, 27, 64].

Why should these people be so vulnerable? The present study has shown that neither the time of onset of hearing loss (pre- or post-lingual) nor level of hearing loss (deafness or partial deafness) explained why their level of global self-esteem was so low. Interestingly, earlier similar studies in the DHH population have shown no correlation between degree of hearing loss and psychological distress [27, 64, 75, 78]. However, people with profound hearing loss, especially if it occurred post-lingually, had a lower level of global self-esteem than the general population. On the other hand, individuals with partial deafness and who use a CI had a global self-esteem similar to the general population. This might be due to them having had a positive experience with their CI, as an earlier study of partial deafness subjects who were not CI users showed that their psychological functioning was generally poorer [67]. We also found that global self-esteem did not depend on whether subjects relied on a CI alone or on a CI in one ear and a HA in the other. The duration of experience with a CI did not seem to affect global self-esteem, even though the subjects were well-experienced CI users (the average length of CI use was 4.2 years).

The crux of the matter appears to be the attitude of the user towards their CI. Our results show that from the variables associated with hearing loss and CI use, the only highly significant predictor of high self-esteem was a high level of satisfaction with their CI. Of course, this is only a correlation, and it may be that those with higher self-esteem are likely to be more satisfied with their CI. One could imagine this may be because they perform tasks better, are more persistent, more active, or bring a more positive attitude than those with lower self-esteem [2,25,79]. It has been shown, for example, that people with higher self-esteem generally have a more positive attitude towards their hearing impairment, and more easily accept their hearing loss [80]. Alternatively, people with higher global self-esteem may be able to cope more effectively with the drawbacks associated with a CI (e.g., distorted or electronic-like sounds). In other studies we have done (although this time only on post-lingual CI users), the degree of global self-esteem also correlated very well with the degree of satisfaction [8].

Turning now to the difference between pre- and post-lingually deafened subjects, our results showed that the post-lingually deafened had the lowest self-esteem of all CI users. For some reason, they were most affected by the negative, devastating effects–the “trauma” as a number of our subjects called it–of profound or total loss of hearing [19,44–45,48,81]. Therefore, this group of patients should be a priority for professional psycho-educational, psychological, and psychotherapeutic interventions aimed at maintaining their self-esteem. Similarly, other activities, such as joining informal support groups for post-lingually deaf people, might be beneficial in terms of providing social or emotional support. The psychological state of the post-lingually deaf [8,44] is still not being adequately addressed by hearing and speech rehabilitation specialists. The stress induced by loss of hearing and its effects on life often fail to go away with time, and those who cannot deal with it need professional support.

Although the CI “works” in terms of acting as a hearing prosthesis and enabling perception of surroundings sounds and speech [17,21,71,82], it is not as good as the natural hearing. Its electronic-like sounds are difficult to interpret, particularly for the post-lingually deaf, and can be a reminder of what they have lost. Frequently, a CI is not able, in itself, to rebuild the self-esteem of those affected by hearing loss. Moreover, the remedy provided by a CI takes a considerable time: first the surgery, then learning to interpret the novel sounds it provides, and finally to understand speech and achieve communication. A CI is not just a medical intervention involving the ear, but requires a total engagement and reorientation of a person's whole approach to life [83].

On the other hand, global self-esteem in other CI users–people with pre-lingual profound deafness or with partial deafness of pre- or post-lingual origin–was comparable to the self-esteem of the general population. This indicates that, among other things, a CI can certainly help these people achieve a good quality of life and high self-esteem. Motivation and a positive attitude are therefore essential to overcome the inherent limitations of a CI. In this respect it should be remembered that people who volunteer for CI surgery may already have higher than average self-esteem and a stronger desire to improve their quality of life, particularly as it relates to health and hearing [25]. Again, it should be recognized that our findings are only correlations, and cause and effect can only be guessed.

### Self-esteem and sociodemographic variables

Among the group of sociodemographic variables it was found that being in a relationship was the biggest factor in elevating a CI user’s level of self-esteem. However, among the study group only 47.5% were in a relationship, which is appreciably less than in the general Polish population, about 62% [84]. The lower proportion may be a reflection of the prevalence of psychosocial difficulties reported in pre-lingually deaf children and adolescents, including reduced social competence and difficulties making friends [85–87]. As for marriage and partnerships, deaf people generally have greater difficulty establishing intimacy compared to hearing persons [7]. At the same time, for people with post-lingual deafness, hearing loss and its consequences often have negative effects on existing relationships [6,19,45,47–48]. At a more general level, recent studies in the wider population have shown that good quality relationships are conducive to self-esteem, and higher self-esteem is also likely to be responsible for creating closer relationships [10,88–89].

We found that higher levels of education was another important factor promoting self-esteem. The same sort of relationship was obtained in Carter's 2016 study of self-esteem in deaf people of varying degrees and etiologies (although no data was shown on how many subjects used a CI) [9]. A similar trend has been found in the general population as well [10,13–14]. Naturally, an association between self-esteem and higher education might also imply that people with higher self-esteem are generally more likely to work towards a higher education, and the same trend may also be evident in the DHH population.

In our study, male CI users tended to have significantly higher self-esteem than females, with the men having a level of self-esteem not much different to men in the general population. Female CI users not only had lower self-esteem than men, but also lower than that of normally hearing women from the general population. Most self-esteem studies of deaf people do not show such a difference between the genders. On the other hand, some studies of the general population do show that men tend to have higher self-esteem than women [10,14].

Our findings showed that occupational activity (or learning) also appeared to be linked to global self-esteem, although it should be stressed that this sociodemographic variable had borderline predictive properties. However, in general, CI users who were professionally active, or studying, had higher levels of self-esteem. In our group, those who were actively working or studying were 59.2% of the total, which is similar to the percentage of those of working age in Poland– 56.1% in 2016 [90]. The relatively high occupational activity of our study group might be related to the apparent beneficial role of CIs, since other studies have shown a generally lower employment rate of both pre- and post-lingually deaf people compared to the hearing population [47, 91–94].

### RSES structure

Based on the results of the CFA, we can say that the two-way model (in which positive and negative items are placed in separate groups) was not confirmed with respect to the RSES results obtained in our study for DHH CI users. Perhaps this result reveals the specificity of the study population, which is expressed by a one-factor solution.

### Limitations and proposals for the future

These studies are among the first to address the self-esteem of the DHH (including some who were partially deaf) who had used a CI from adulthood. However, the relatively small sample size is a limiting factor. In the future, it would be interesting to look at adult CI users who received a CI in early childhood and grew up with a CI. There are still only a few studies that have examined long-term outcomes based on reports from the patients themselves [59,95]. Of course, global self-esteem would be one such long-term outcome.

An interesting population are those DHH people who are CI users but identify with the Deaf community, an important linguistic and cultural minority [35,37,39,43,54,64]. Sign language is their first language, and their psychosocial development, including self-esteem, continue to be affected by the Deaf community. To take account of the special role of sign language, it would seem appropriate–irrespective of whether a person uses a CI or not–to adapt RSES to Polish Sign Language. In the original RSES, the majority of items assume high language competency. It would help if RSES were modified linguistically to make it accessible to those members of the DHH population who have limited competency in either spoken or sign language. A modified version would allow study of the self-esteem of graduates of special schools for the DHH, especially those who have serious limitations in spoken or sign language [32‒35].

A limitation of the presented study relates to its cross-sectional design and the purely correlational approach. This means we cannot say for sure that use of a CI contributes to raising global self-esteem, although the two factors are at least correlated. It seems reasonable to assume that the CI, through its ability to provide a form of hearing (and the associated nonaudiological benefits), will support psychosocial processes such as language development and interaction with the environment, and therefore help improve the quality of life of DHH people. In doing that, it appears to contribute to their global self-esteem.

## Conclusion

Self-esteem is important, especially for populations at risk of mental health problems, which is the DHH population. Despite all the unquestionable benefits of a CI as a hearing prosthesis, not all CI users deal completely with the trauma of hearing loss. Thus, as de Graaf and Bijl (2002) postulated more than 15 years ago [27], every effort should be made to psychologically support DHH people in accepting their deafness or hearing loss, and to raise and improve their global self-esteem–which will in turn reduce their mental distress. All interventions which aim to increase self-esteem–whether by psychological, psychoeducational, or psychotherapeutic means–help improve people’s mental health and well-being [10]. There is no reason to think it is different when it comes to the DHH population.

## Supporting information

S1 File Individual data for all subjects. (XLSX)

## References

[1] Rosenberg M. Society and adolescent self-image Princeton, NJ: Princeton University Press; 1965.

[2] Dzwonkowska I, Łachowicz-Tabaczek K, Łaguna M. Samoocena i jej pomiar. Polska adaptacja skali SES M. Rosenberga. [Self-esteem and its measures Polish adaptation of the Rosenberg’s Self-Esteem Scale SES]. Warszawa: Pracownia Testów Psychologicznych; 2008. Polish.

[3] Alessandri G, Vecchione M, Eisenberg N, Łaguna M. On the factor structure of the Rosenberg (1965) General Self-Esteem Scale. Psychol Assess. 2015; 27(2):621–35. 10.1037/pas0000073 25580614

[4] Crowe TV. Self-esteem scores among deaf college students: an examination of gender and parents' hearing status and signing ability. J Deaf Stud Deaf Educ. 2003; 8: 199–206. 10.1093/deafed/eng003 15448068

[5] Hintermair M. Self-esteem and satisfaction with life of deaf and hard-of-hearing people–A resource-oriented approach to identity work. J Deaf Stud Deaf Educ. 2008;13(2): 278–300. 10.1093/deafed/enm054 17971343

[6] Kashubeck-West S, Meyer J. The well-being of women who are late deafened. J Couns Psychol. 2008; 55:463–72. 10.1037/a0013619 22017553

[7] Levinger M, Ronen T. The link among self-esteem, differentiation, and spousal intimacy in deaf and hearing adults. J Soc Work Disabil Rehabil. 2010; 9:27–52. 10.1080/15367100903526120 20391079

[8] Kobosko J, Jedrzejczak WW, Pilka E, Pankowska A, Skarzynski H. Satisfaction with cochlear implants in postlingually deaf adults and its nonaudiological predictors: psychological distress, coping strategies, and self-esteem. Ear Hear. 2015; 36:605–18. 10.1097/AUD.0000000000000179 25973692

[9] Carter MJ, Mireles DC. Exploring the relationship between deaf identity verification processes and self-esteem. Identity. 2016; 16:102–14.

[10] Orth U. The lifespan development of self-esteem In: Specht J, editor. Personality development across the lifespan. London, UK: Elsevier; 2017 p. 181–195.

[11] von Soest T, Wichstrøm L, Kvalem IL. The development of global and domain-specific self-esteem from age 13 to 31. J Pers Soc Psychol. 2016; 110: 592–608. 10.1037/pspp0000060 26167796

[12] Mund M, Finn C, Hagemeyer B, Zimmermann J, Neyer FJ. The dynamics of self‐esteem in partner relationships. Eur J Pers. 2015; 29:235–49. 10.1002/per.1984

[13] Sinclair SJ, Blais MA, Gansler DA, Sandberg E, Bistis K, LoCicero A. Psychometric properties of the Rosenberg Self-Esteem Scale: Overall and across demographic groups living within the United States. Eval Health Prof. 2010; 33:56–80. 10.1177/0163278709356187 20164106

[14] Szpitalak M, Polczyk R. Samoocena. Geneza, struktura, funkcje i metody pomiaru [Self-esteem. Genesis, structure, functions and measurement methods] Kraków: Wydawnictwo Uniwersytetu Jagiellońskiego; 2015. Polish.

[15] Bureau International d’ Audiophonologie (BIAP). Recommendation BIAP 02/1bis. Classification audiométrique des déficiences auditives. [Internet]. Available from: http://www.biap.org/en/recommendations/65-ct-2-classification-des-surdites/5-recommandation-biap-021-bis. French.

[16] Skarzynski H, Lorens A, Piotrowska A, Skarzynski PH. Hearing preservation in partial deafness treatment. Med Sci Monit. 2010 11;16(11):CR555–62. 20980961

[17] Lazard DS, Vincent C, Venail F, Van de Heyning P, Truy E, Sterkers O et al Pre-, per- and postoperative factors affecting performance of postlinguistically deaf adults using cochlear implants: a new conceptual model over time. PLoS One. 2012;7(11):e48739 10.1371/journal.pone.0048739 23152797 PMC3494723

[18] Bosco E, Nicastri M, Ballantyne D, Viccaro M, Ruoppolo G, Maddalena AI et al. Long term results in late implanted adolescent and adult CI recipients. Eur Arch Otorhinolaryngol. 2013; 270: 2611–20. 10.1007/s00405-012-2264-4 23179930

[19] Mäki-Torkko EM, Vestergren S, Harder H, Lyxell B. From isolation and dependence to autonomy–expectations before and experiences after cochlear implantation in adult cochlear implant users and their significant others. Disabil Rehab. 2015; 37: 541–47. 10.3109/09638288.2014.935490 24989065

[20] Ramos-Macías Á, Falcón González JC, Borkoski-Barreiro SA, Ramos de Miguel Á, Batista DS, Pérez Plasencia D. Health-related quality of life in adult cochlear implant users: A descriptive observational study. Audiol Neurotol, 2016; 21(Suppl. 1): 36–42. 10.1159/000448353 27806363

[21] Capretta NR, Moberly AC. Does quality of life depend on speech recognition performance for adult cochlear implant users? Laryngoscope, 2016; 126(3): 699–706. 10.1002/lary.25525 26256441

[22] Skarzyński H, Janczewski G, Niemczyk K, Kochanek K, Geremek A, Klasek O. Pierwszy implant ślimakowy w Polsce [First cochlear implant in Poland]. Otolaryngol Pol. 1993; 47:427–34. Polish. 8259292

[23] Skarżyński H. Wszczep ślimakowy u osoby dorosłej z głuchotą prelingwalną [Cochlear implant in the adult person with prelingual deafness]. Otolaryngol Pol. 1994; 48(Supl. 15): 152–9. Polish.8028906

[24] Skarzynski H, Lorens A, Piotrowska A. A new method of partial deafness treatment. Med Sci Monit. 2003; 9:CS20–24. 12709676

[25] Baumeister RF, Campbell JD, Krueger JI, Vohs KD. Does high self-esteem cause better performance, interpersonal success, happiness, or healthier lifestyles? Psychol Sci Publ Interest. 2003; 4:1–44. 10.1111/1529-1006.01431 26151640

[26] Orth U, Robins RW, Widaman KF. Life-span development of self-esteem and its effects on important life outcomes. J Pers Soc Psychol. 2012; 102(6):1271–88. 10.1037/a0025558 21942279

[27] de Graaf R, Bijl RV. Determinants of mental distress in adults with a severe auditory impairment: differences between prelingual and postlingual deafness. Psychosom Med. 2002; 64:61–70. 11818587 10.1097/00006842-200201000-00009

[28] Huber M, Burger T, Illg A, Kunze S, Giourgas A, Braun, et al A. Mental health problems in adolescents with cochlear implants: peer problems persist after controlling for additional handicaps. Front Psychol. 2015; 6:953 10.3389/fpsyg.2015.00953 26236251 PMC4502340

[29] Zalewska M. Dziecko w autoportrecie z zamalowana twarzą. Psychiczne mechanizmy zaburzeń rozwoju tożsamości dziecka głuchego i dziecka z opóźnionym rozwojem mowy [Self-portrait of a child with the face painted over. Psychological mechanisms of identity development disorders of deaf children and children with language delay] Warszawa: J. Santorski & CO Wydawnictwo; 1998.

[30] Black PA, Glickman NS. Demographics, psychiatric diagnoses, and other characteristics of North American deaf and hard-of-hearing inpatients. J Deaf Stud Deaf Educ. 2006; 11:303–21. 10.1093/deafed/enj042 16687730

[31] Quittner AL, Barker DH, Cruz I, Snell C, Grimley ME, Botteri M, et al Parenting stress among parents of deaf and hearing children: Associations with language delays and behavior problems. Parent Sci Pract. 2010; 10:136–55. 10.1080/15295190903212851 20607098 PMC2895932

[32] Krakowiak K. Dar języka. Podręcznik metodyki wychowania językowego dzieci i młodzieży z uszkodzeniami narządu słuchu [A gift of language. Course book of methodology of language education for children and youths with hearing disorders] Lublin: Wydawnictwo Katolickiego Uniwersytetu Lubelskiego; 2012. Polish.

[33] Kobosko J. The mother-child relationship and language development disorders: Studies of deaf adolescent children of hearing parents In: Estrada Aranda B, Sleeboom-van Raaij I, editors. Mental health services for deaf people. Treatment, advances, opportunities, and challenges. Washington: Gallaudet University Press; 2015 p. 193–214.

[34] Marschark M, Machmer E, Spencer LJ, Borgna G, Durkin A, Convertino C. Language and psychosocial functioning among deaf learners with and without cochlear implants. J Deaf Stud Deaf Educ. 2018; 23: 28–40. 10.1093/deafed/enx035 28977414 PMC5873730

[35] Wojda P. Transmission of Polish Sign Systems In: Brentari D, editor. Sign languages: A Cambridge language survey. Cambridge, United Kingdom: Cambridge University Press; 2010 p. 131–47.

[36] Kobosko J, Zalewska M. Maternal identity of hearing mothers of deaf adolescents. Empirical studies–an interpersonal approach. Volta Rev. 2011; 111:39–59.

[37] Lane H. The mask of benevolence Disabling the deaf community. New York: Vintage Books, Division of Random House, Inc; 1993.

[38] Jambor E, Elliott M. Self-esteem and coping strategies among deaf students. J Deaf Stud Deaf Educ. 2005; 10: 63–81. 10.1093/deafed/eni004 15585749

[39] Leigh IW. A lens on deaf identities Perspectives on deafness. Oxford: Oxford University Press; 2009.

[40] Orth U. The family environment in early childhood has a long-term effect on self-esteem: A longitudinal study from birth to age 27 years. J Pers Soc Psychol. 2018; 114: 637–655. 10.1037/pspp0000143 28182449

[41] Brice PJ, Adams EB. Developing a concept of self and other: risk and protective factors In: Zand DH, Pierce KJ, editors. Resilience in deaf children. New York, Dordrecht, Heidelberg, London: Springer; 2011 p. 115–37.

[42] Bat-Chava Y. Antecedents of self-esteem in deaf people: A meta-analytic review. Rehabil Psychol. 1993; 38:221–34. 10.1037/h0080303

[43] Tomaszewski P, Sak M. Is it possible to educate deaf children bilingually in Poland? In: Olpińska-Szkiełko M, Bertelle L, editors. Zweisprachigkeit und Bilingualer Unterricht. Frankfurt: Peter Lang Press; 2014 p. 129–49.

[44] Du Feu M, Fergusson K. Sensory impairment and mental health. Adv Psychiatr Treat. 2003; 9: 95–103.

[45] du Feu M. Deafened people In: du Feu M, Chovaz C. Mental health and deafness. Oxford: Oxford University Press; 2014 p. 209–15.

[46] Orth U, Luciano EC. Self-esteem, narcissism, and stressful life events: Testing for selection and socialization. J Pers Soc Psychol. 2015;109:707–21. 10.1037/pspp0000049 26011661

[47] Monzani D, Galeazzi GM, Genovese E, Marrara A, Martini A. Psychological profile and social behaviour of working adults with mild or moderate hearing loss. Acta Otorhinolaryngol Ital. 2008; 28: 61–66. 18669069 PMC2644978

[48] Meyer JM, Kashubeck-West S. Well-being of individuals with late-deafness. Rehabil Psychol. 2013; 8(2):124–36. 10.1037/a0032197 23713725

[49] Kobosko J. Tożsamość macierzyńska matek słyszących młodzieży głuchej i jej znaczenie dla rozwoju osobowej tożsamości tej młodzieży [Maternal identity of hearing mothers of deaf adolescents and its significance for the development of young people’s personal identity] [dissertation] Warszawa: Uniwersytet Warszawski; 2007. Polish.

[50] Dryżałowska G. Integracja edukacyjna a integracja społeczna Satysfakcja z życia osób niedosłyszących [Educational and social integration. Satisfaction with life of people with hearing loss]. Warszawa: Wydawnictwa Uniwersytetu Warszawskiego; 2016. Polish.

[51] Zaborniak-Sobczak M. Odpowiedzialność młodzieży z wadą słuchu [Responsibility of adolescents with hearing loss] Rzeszów: Wydawnictwo Uniwersytetu Rzeszowskiego; 2014. Polish.

[52] Kobosko J. Spostrzeganie wsparcia emocjonalnego a jakość relacji z innymi ludźmi u osób z głuchotą prelingwalną [Perception of emotional support vs. interpersonal relations of prelingually deaf adults using cochlear implant in adulthood] In: Parys K, Pasteczka M, Sikorski J, editors. Teoria i praktyka oddziaływań profilaktyczno-wspierających rozwój osób z niepełnosprawnością –konteksty indywidualne i środowiskowe [The theory and praxis of preventive and supportive activities supporting development of people with disabilities–individual and environmental contexts], t. 4.2. Kraków: Wydawnictwo Uniwersytetu Pedagogicznego; 2017 p. 221–237. Polish.

[53] Kossewska J. Studies on deafness in an ecological system context Kraków: Wydawnictwo JAK; 2016.

[54] Leigh IW, Andrews JF. Deaf people in society: Evolving perspectives in psychology, sociology, and education 2nd ed New York, London: Routledge Taylor and Francis Group; 2017.

[55] Most T, Shrem H, Duvdevani I. Cochlear implantation in late-implanted adults with prelingual deafness. Am J Otolaryngol. 2010; 31(6):418–23. 10.1016/j.amjoto.2009.07.002 20015795

[56] van Gent T, Goedhart AW, Knoors HE, Westenberg PM, Treffers PD. Self-concept and ego development in deaf adolescents: a comparative study. J Deaf Stud Deaf Educ. 2012;17:333–51. 10.1093/deafed/ens002 22351698

[57] Wallis D, Musselman C, MacKay S. Hearing mothers and their deaf children: The relationship between early, ongoing mode match and subsequent mental health functioning in adolescence. J Deaf Stud Deaf Educ. 2004; 9:2–14. 10.1093/deafed/enh014 15304398

[58] Leigh IW, Maxwell-McCaw D, Bat-Chava Y, Christiansen JB. Correlates of psychosocial adjustment in deaf adolescents with and without cochlear implants: A preliminary investigation. J Deaf Stud Deaf Educ. 2009;14:244–59. 10.1093/deafed/enn038 18854552

[59] van Gurp S. Self-concept of deaf secondary school students in different educational settings. J Deaf Stud Deaf Educ. 2001; 6:54–69. 10.1093/deafed/6.1.54 15451863

[60] Mejstad L, Heiling K, Svedin CG. Mental health and self-image among deaf and hard of hearing children. Am Ann Deaf. 2009;153:504–15. 19350957 10.1353/aad.0.0069

[61] Bat-Chava Y. Diversity of deaf identities. Am Ann Deaf. 2000; 145(5): 420–428. 11191821 10.1353/aad.2012.0176

[62] Cornell SL, Lyness KP. Therapeutic implications for adolescent deaf identity and self-concept. J Fam Ther. 2005; 16(3): 31–49.

[63] Maxwell-McCaw D. Acculturation and psychological well-being in Deaf and hard of hearing people [dissertation] Washington, DC: The George Washington University; 2001.

[64] Fellinger J, Holzinger D, Pollard R. Mental health of deaf people. Lancet. 2012; 379:1037–44. 10.1016/S0140-6736(11)61143-4 22423884

[65] Fellinger J, Holzinger D, Gerich J, Goldberg D. Mental distress and quality of life in the hard of hearing. Acta Psychiatr Scand. 2007; 115:243–45. 10.1111/j.1600-0447.2006.00976.x 17302625

[66] Van Gent T, Goedhart AW, Treffers PD. Self‐concept and psychopathology in deaf adolescents: preliminary support for moderating effects of deafness‐related characteristics and peer problems. J Child Psychol Psychiatry. 2011; 52: 720–728. 10.1111/j.1469-7610.2011.02392.x 21418064

[67] Cieśla K, Lewandowska M, Skarżyński H. Health-related quality of life and mental distress in patients with partial deafness: preliminary findings. Eur Arch Otorhinolaryngol. 2016; 273(3):767–76. 10.1007/s00405-015-3713-7 26242252 PMC4762916

[68] Chung J, Chueng K, Shipp D, Friesen L, Chen JM, Nedzelski JM, Lin VY. Unilateral multi-channel cochlear implantation results in significant improvement in quality of life. Otol Neurotol. 2012; 33(4): 566–71. 10.1097/MAO.0b013e3182536dc2 22569148

[69] Brüggemann P, Szczepek AJ, Klee K, Gräbel S, Mazurek B, Olze H. In patients undergoing cochlear implantation, psychological burden affects tinnitus and the overall outcome of auditory rehabilitation. Front Hum Neurosci. 2017; 11:226 10.3389/fnhum.2017.00226 28529479 PMC5418338

[70] Kobosko J, Pankowska A, Olszewski Ł, Geremek-Samsonowicz A, Skarżyński H. Subiektywna i obiektywna ocena korzyści z implantu ślimakowego u osób dorosłych z częściową głuchotą o początku prelingwalnym [Subjective and objective assessment of cochlear implant benefit in adults with the prelingual onset partial deafness]. Now Audiofonol. 2017; 6(4):31–42. Polish. doi: 10.17431/1002752

[71] Solnica J, Kobosko J, Pankowska A, Zgoda M, Skarżyński H. Efektywność treningu słuchowego osób z częściową głuchotą po wszczepieniu implantu ślimakowego w ocenie pacjentów i logopedów [Effectiveness of the auditory training in patients with the partial deafness after cochlear implantation in the assessment of patients and speech therapists]. Now Audiofonol. 2012; 1(1):31–37. Polish.

[72] Schmitt DP, Allik J. Simultaneous administration of the Rosenberg Self-Esteem Scale in 53 nations: exploring the universal and culture-specific features of global self-esteem. J Pers Soc Psychol. 2005; 89:623–42. 10.1037/0022-3514.89.4.623 16287423

[73] Hu LT, Bentler PM. Cutoff criteria for fit indexes in covariance structure analysis: Conventional criteria versus new alternatives. Struct Equ Model. 1999; 6:1–55.

[74] Bat-Chava Y. Group identification and self-esteem of deaf adults. Pers Soc Psychol Bull. 1994; 20:494–502.

[75] Tambs K. Moderate effects of hearing loss on mental health and subjective well-being: From the Nord-Trøndelag hearing loss study. Psychosom Med. 2004; 66:776–82. 10.1097/01.psy.0000133328.03596.fb 15385706

[76] Lu A, Hong X, Yu Y, Ling H, Tian H, Yu Z, et al Perceived physical appearance and life satisfaction: A moderated mediation model of self-esteem and life experience of deaf and hearing adolescents. J Adolesc. 2015; 39:1–9. 10.1016/j.adolescence.2014.11.005 25540861

[77] Theunissen SCPM, Netten AP, Rieffe C, Briaire JJ, Soede W, Kouwenberg M, et al Self-esteem in hearing-impaired children: The influence of communication, education, and audiological characteristics. PLoS ONE; 9(4):e94521 10.1371/journal.pone.0094521 24722329 PMC3983202

[78] Fellinger J, Holzinger D, Sattel H, Laucht M, Goldberg D. Correlates of mental health disorders among children with hearing impairments. Dev Med Child Neurol. 2009; 51:635–41. 10.1111/j.1469-8749.2008.03218.x 19627335

[79] Baumeister RF, Smart L, Boden JM. Relation of threatened egotism to violence and aggression: the dark side of high self-esteem. Psychol Rev. 1996;103:5–33. 8650299 10.1037/0033-295x.103.1.5

[80] Saunders GH, Cienkowski KM. Refinement and psychometric evaluation of the attitudes toward loss of hearing questionnaire. Ear Hear. 1996;17:505–19. 8979038 10.1097/00003446-199612000-00006

[81] Hallam R, Ashton P, Sherbourne K, Giley L. Acquired profound hearing loss: Mental health and other characteristics of a large sample. Int J Audiol. 2006; 45:715–723. 10.1080/14992020600957335 17132560

[82] McRackan TR, Bauschard M, Hatch JL, Franko-Tobin E, Droghini HR, Nguyen SA, Dubno JR. Meta-analysis of quality-of-life improvement after cochlear implantation and associations with speech recognition abilities. Laryngoscope. 2018; 128: 982–990. 10.1002/lary.26738 28731538 PMC5776066

[83] Finlay L, Molano-Fisher P. ‘Transforming’self and world: a phenomenological study of a changing lifeworld following a cochlear implant. Med Health Care Philos. 2008 9;11(3):255–67. 10.1007/s11019-007-9116-9 18060574

[84] Główny Urząd Statystyczny (Departament Badań Demograficznych, Departament Pracy, Departament Handlu i Usług). Raport z wyników. Narodowy Spis Powszechny Ludności i Mieszkań 2011 [Central Statistical Office of Poland (Department of Demographic Studies, Department of Work, Department of Trade and Services) (2012). Report of Results. National Census of Population and Housing 2011. Warszawa, Zakład Wydawnictw Statystycznych; 2012. http://www.stat.gov.pl/

[85] Suaréz M. Promoting social competence in deaf students: The effect of an intervention program. J Deaf Stud Deaf Educ. 2000; 5:323–33. 10.1093/deafed/5.4.323 15454498

[86] Most T, Ingber S, Heled-Ariam E. Social competence, sense of loneliness, and speech intelligibility of young children with hearing loss in individual inclusion and group inclusion. J Deaf Stud Deaf Educ. 2012;17:259–72. 10.1093/deafed/enr049 22186369

[87] Hoffman MF, Cejas I, Quittner AL, CDaCI Investigative Team. Comparisons of longitudinal trajectories of social competence: Parent ratings of children with cochlear implants versus hearing peers. Otol Neurotol. 2016; 37:152–9. 10.1097/MAO.0000000000000938 26719958 PMC4712083

[88] Erol RY, Orth U. Development of self-esteem and relationship satisfaction in couples. Two longitudinal studies. Dev Psychol. 2014; 50:2291–303. 10.1037/a0037370 24999764

[89] Erol RY, Orth U. Self-esteem and the quality of romantic relationships. Eur Psychol. 2016; 21:274–83.

[90] Rynek pracy. [Internet]. Available from: http://rynekpracy.org/x/902899. Polish.

[91] Punch R, Hyde M, Power D. Career and workplace experiences of Australian university graduates who are deaf or hard of hearing. J Deaf Stud Deaf Educ. 2007; 12:504–17. 10.1093/deafed/enm011 17470440

[92] Punch R. Employment and adults who are deaf or hard of hearing: Current status and experiences of barriers, accommodations, and stress in the workplace. Am Ann Deaf. 2016;161:384–97. 10.1353/aad.2016.0028 27477043

[93] Williams KC, Falkum E, Martinsen EW. Fear of negative evaluation, avoidance and mental distress among hearing-impaired employees. Rehabil Psychol. 2015; 60: 51–58. 10.1037/rep0000028 25621920

[94] Emmett SD, Francis HW. The socioeconomic impact of hearing loss in US adults. Otol Neurotol. 2015; 36:545–50. 10.1097/MAO.0000000000000562 25158616 PMC4466103

[95] Spencer LJ, Tomblin JB, Gantz BJ. (2012), Growing up with a cochlear implant: education, vocation, and affiliation. J Deaf Stud Deaf Educ. 2012;17:483–98. 10.1093/deafed/ens024 22949609 PMC3459294

